# Whole of Systems Trial of Prevention Strategies for Childhood Obesity: WHO STOPS Childhood Obesity

**DOI:** 10.3390/ijerph13111143

**Published:** 2016-11-16

**Authors:** Steven Allender, Lynne Millar, Peter Hovmand, Colin Bell, Marj Moodie, Rob Carter, Boyd Swinburn, Claudia Strugnell, Janette Lowe, Kayla de la Haye, Liliana Orellana, Sue Morgan

**Affiliations:** 1 Global Obesity Centre (GLOBE), World Health Organization Collaborating Centre for Obesity Prevention, Faculty of Health, Deakin University, Melbourne 3125, Australia; lynne.millar@deakin.edu.au (L.M.); colin.bell@deakin.edu.au (C.B.); marj.moodie@deakin.edu.au (M.M.); boyd.swinburn@auckland.ac.nz (B.S.); claudia.strugnell@deakin.edu.au (C.S.); 2 Brown School’s Social System Design Lab, Washington University, St Louis, MO 63130, USA; phovmand@wustl.edu; 3 School of Medicine, Faculty of Health, Deakin University, Melbourne 3125, Australia; 4 Deakin Health Economics, Centre for Population Health Research, Faculty of Health, Deakin University, Melbourne 3125, Australia; rob.carter@deakin.edu.au; 5 Population Nutrition and Global Health, University of Auckland, Auckland, NZ 1142, USA; 6 Southern Grampians and Glenelg Primary Care Partnership, Hamilton, NZ 3300, USA; Janette.Lowe@wdhs.net; 7 Department of Preventive Medicine, University of Southern California, Los Angeles, CA 90033, USA; delahaye@usc.edu; 8 Biostatistics Unit, Faculty of Health, Deakin University, Melbourne 3125, Australia; l.orellana@deakin.edu.au; 9 Victorian Department of Health and Human Services, Geelong 3220, Australia; sue.morgan@dhhs.vic.gov.au

**Keywords:** community prevention, childhood obesity, systems thinking, cluster randomized controlled trial, social network analysis, economic analysis, anthropometry, obesogenic behaviours

## Abstract

*Background*: Community-based initiatives show promise for preventing childhood obesity. They are characterized by community leaders and members working together to address complex local drivers of energy balance. *Objectives*: To present a protocol for a stepped wedge cluster randomized trial in ten communities in the Great South Coast Region of Victoria, Australia to test whether it is possible to: (1) strengthen community action for childhood obesity prevention, and (2) measure the impact of increased action on risk factors for childhood obesity. *Methods:* The WHO STOPS intervention involves a facilitated community engagement process that: creates an agreed systems map of childhood obesity causes for a community; identifies intervention opportunities through leveraging the dynamic aspects of the system; and, converts these understandings into community-built, systems-oriented action plans. Ten communities will be randomized (1:1) to intervention or control in year one and all communities will be included by year three. The primary outcome is childhood obesity prevalence among grade two (ages 7–8 y), grade four (9–10 y) and grade six (11–12 y) students measured using our established community-led monitoring system (69% school and 93% student participation rate in government and independent schools). An additional group of 13 external communities from other regions of Victoria with no specific interventions will provide an external comparison. These communities will also allow us to assess diffusion of the intervention to control communities during the first three years of the trial. *Conclusion*: This trial will test effectiveness, over a five-year period, of community-owned, -supported and -led strategies designed to address complex and dynamic causes of childhood obesity.

## 1. Introduction

Obesity is a major determinant of Type 2 diabetes, coronary heart disease, and cancer. In Australia, obesity costs the health system more than $21 billion annually [1] and more than one-quarter (28%) of Australian children are overweight or obese [2]. The size and scope of the burden, together with overweight youth having a 70% chance of becoming obese adults [3], make the case for prevention compelling. Children are a critical focus for prevention because they are so susceptible to the environment and because treatment is difficult. Childhood obesity has been recognized as a priority in the WHO’s Global 2013–2020 Action Plan for the Prevention and Control of Non Communicable Disease [4] and recommendations have been provided to countries on ending childhood obesity [5].

Recent Cochrane meta-analysis of childhood obesity interventions showed an overall benefit for community-based interventions among primary school-aged children [6]. The 55 studies reviewed evaluated mainly small, discrete interventions delivered over the short term and not “scaled up” to population levels. Several successful community-based studies [7,8,9] showed that improving broader system determinants (e.g., community capacity), strongly predicts the degree of reduction in childhood obesity [10]. The 2015 Lancet Obesity Series [11] identified the challenge for community-based childhood obesity prevention initiatives as the creation of sustained, large-scale interventions that work at multiple levels [12].

Several attempts have been made or are underway to work at population scale in Australia (e.g., Healthy Together Victoria), New Zealand [13] and England [14]. These population-level interventions begin by fostering a shared understanding of the systemic determinants of non-communicable disease and asking how existing systems can be strengthened or new systems created to better promote health and prevent disease [15]. One of the clear messages from previously successful trials is that building community capacity to apply systems thinking is critical [10]. Systems thinking is characterized by attempts to identify the most important cause and effect relationships within a specific system boundary that create feedback and so amplify or stabilise change across a system. System dynamics, an approach to systems thinking, explicitly seeks to create informal maps and formal simulation models of this dynamic complexity [16]. While many more traditional interventions have focused on linear cause-and-effect relationships, systems interventions focus on non-linear relationships (e.g., tipping points), feedback where a “causal” variable might in turn be impacted by an “outcome variable”, and complexity in the multilevel factors involved, as well as multiplex relationships among these factors.

New methods are required that facilitate communities’ and researchers’ ability to measure the components and processes of relevant systems (i.e., systems that impact childhood obesity in their community), to map and understand these systems, and to use systems data and models in real time to implement change and make ongoing improvements at multiple levels. Similarly, new methods are required to integrate economic analysis into systems approaches so that cost-effectiveness can be measured.

In this paper, we present the protocol for the Whole of Systems Trial of Prevention Strategies for childhood obesity (WHO STOPS childhood obesity) trial. This trial has grown out of strong partnerships between researchers and community leaders in the Great South Coast Region of Victoria (GSCRV), Australia. Shared work in preparation for this study has included creation of: (1) a sustainable and high participatory obesity-monitoring system; and (2) whole of community systems measures and interventions.

### 1.1. A Sustainable Monitoring System

In 2015, a childhood obesity-monitoring system was established across six local government areas in South-Western Victoria [17]. We used locally sourced competitive funding to conduct training, manage data collection, conduct analysis and support an in-kind contribution from health services, local government and schools to conduct data collection. Anthropometric and behavioural data were collected from 90% of children in 48 of the 84 primary schools in the GSCRV. Partners have committed to provide in-kind support to collect these data again in 2017 and 2019.

### 1.2. Whole of Community Systems Measures and Interventions

The researchers and community leaders have developed and piloted a range of systems action tools and techniques to build community capacity and ownership of efforts to apply system thinking to community-wide childhood obesity prevention.

This paper sets out a protocol for the WHO STOPS Obesity project designed to bring all these lessons together and test the efficacy and cost-effectiveness of the approach for preventing obesity among primary school-aged children in South-Western Victoria, Australia.

The trial is registered by the Australian New Zealand Clinical Trials Registry (ACTRN12616000980437).

## 2. Methods

This is a cluster randomized stepped wedge trial (two steps) in ten communities. The unit of randomisation and intervention is the community (rather than individual children). Following baseline measurement of all eligible children in all ten communities (2015), five communities will be randomized to the intervention in year one (step one) and the remaining five will be entered into the intervention two years later (step two) (Table 1). Grades two, four and six children in all clusters will be measured in the baseline year (2015), year two (2017) and year four (2019). Data collection every second year reflects a more cost-effective measurement approach and a realistic timeframe to begin to see changes in weight status among intervention populations. Opportunities and funding for longer-term data collection (i.e., a five- and ten-year follow up) will be actively sought in due course.

### 2.1. Study Population

**Figure 1:**
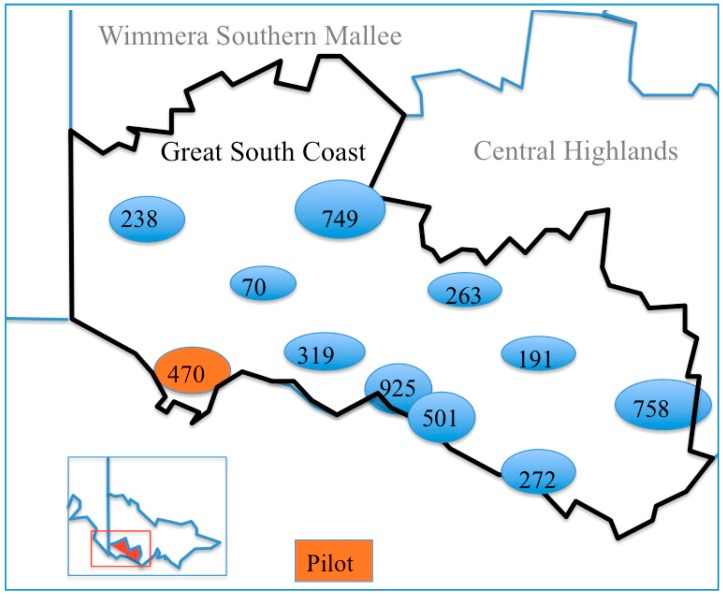
Estimated number of eligible children (grades two, four and six) in study region: *n* presented in shaded bubbles.

The units of observation are all primary school-aged children in grades two, four and six (aged approximately 6 to 12 years) (*n* = 5050) in ten communities of the GSCRV. Clusters, or “natural communities”, are distinct, dispersed population centres agreed by partners based on existing government, health service, and education boundaries (see Figure 1). The ten enrolled clusters will be ranked in order of size, and one community from each quintile will be randomly allocated to intervention at step one. Blinding and allocation concealment is not possible in trials where whole communities are recruited into the design, implementation and evaluation.

This stepped wedge design [18,19] is particularly suited to situations where: randomisation of individuals is not possible (i.e., population level trials); the intervention is expected to be of benefit and unlikely to do any harm (i.e., where previous small-scale interventions have been successful); and, where allocation of communities to a control-only arm would be problematic, unethical, or likely to result in communities refusing to participate [20,21]. The design provides strong analytic qualities, with intervention/control exposure and comparison available both within and across arms [22,23]. The trial described requires intensive support efforts across multiple communities and so it is not feasible to deliver it across all communities simultaneously. It is also designed iteratively so that subsequent communities benefit from lessons learned from the preceding communities.

### 2.2. Intervention

Intensive training and support within each intervention community will be oriented around strengthening WHO systems building blocks [24,25] (leadership, workforce development, resources, intelligence) and the New South Wales capacity-building framework [26] (partners and networks) in community settings. This includes mapping existing systems and using these maps to develop and implement whole of systems change with community members and implementation support to optimize interventions. Our pilot work [27] shows that community members were able to identify multiple systems that impact childhood obesity (examples range from improvements in individual health literacy, changes to school food and physical environments, banning of sugar-sweetened beverages within institutions, and local government regulation for better health) and design interventions that consider non-linearity, feedback and complexity.

The system intervention will be implemented by community members (parents and leaders from local government, education, clubs, health agencies, and businesses) with influence on environments in which children experience the key obesity risk factors. Partners will convene new and existing coalitions of community leaders who have the authority, capacity, and networks to lead systems change across the community. These leaders will form a steering group comprising members who are prepared to prioritize changing community systems to healthier food choices, physical activity and childhood obesity prevention across the intervention design process (Table 2). The process is typically conducted within a six-week period with six monthly reviews to capture systems change.

This systems-oriented intervention is similar to previous community capacity-building interventions [28] but builds on them in a number of critical ways. It adds: (1) a new way of thinking about embedding actions in systems; (2) an explicit picture of current community assets in relation to systems; (3) a process for enhancing community engagement in their own systems; (4) a dynamic logic model that adapts to change rather than a fixed linear model; (5) a planning tool for developing and prioritizing a broader set of community-wide actions; (6) a communications device to engage wider stakeholders; (7) a set of tools for community diagnostic and evaluative measurements; (8) an approach that is scalable and sustainable; (9) economic evaluation that utilizes conventional appraisal alongside newer approaches that capture interactions and dynamic changes through time; and, (10) empirical evaluation of the participating community partners’ social networks, and roles that these social networks play in intervention diffusion and increasing community capacity.

### 2.3. Characterizing and Intervening in Systems

Using Action Research [29] and Knowledge Translation and Exchange frameworks [30], the nature of the intervention is allowed to iterate and develop over time. The following measures will be taken and be fed back to progressive design and implementation support workshops described above.

#### Systems Maps

Group model building (GMB) will be used to develop causal maps (causal loop diagrams) as qualitative representations of the feedback mechanisms and delays of systems driving obesity trends within each community. GMB is a participatory systems science method for engaging stakeholders in the process of developing informal maps and formal models with computer simulation [31]. Central to GMB is the explicit design and facilitation of a sequence of boundary objects that allow all diverse stakeholders to describe dependencies between different elements of systems [32,33,34].

The GMB workshops comprise facilitated activities that provide the skills and techniques necessary for participants to develop system maps using GMB scripts [35,36]. In each community, an evidence-based, community-specific systems logic model will be co-developed and validated by participants and researchers and priority actions formed. Subsequent workshops (six monthly) will identify subsystems changes and modifications to systems. Quantitative data will be collected to describe key variables in the system logic model. Where data are not available, community measures of variable strength and change will be collected. These data will be used for the economic analysis to describe the level and cost of intervention activity in relation to the measured outcome.

### 2.4. Evaluation

#### 2.4.1. External Control Communities

Thirteen additional Victorian communities were selected in the 2014/2015 financial year as comparison (i.e., no intervention) sites [37]. Baseline data was collected in parallel to the baseline for this study from 2561 of 2959 eligible students in Victorian primary schools (response rate = 87%). The outcome measures used were identical to those in the current proposal, collected under an opt-out consent model, and in regions geographically dispersed from the GSCRV. Measures will be repeated in 2016/2017 and 2018/2019 to provide a detailed picture of changes in BMI-z (Body Mass Index z score) and risk factor prevalence outside the GSCRV. This will also provide for some assessment of contamination across areas. We will be able to assess regional differences though not broader efforts. For example, we will be able to assess the effect of different localized health promotion media, though not state-wide or federal coverage.

#### 2.4.2. Primary and Secondary Outcomes

Primary outcome: change in childhood BMI-z and obesity prevalence. Secondary outcomes: change in children’s diet and physical activity (PA) behaviours, community systems, and settings environments for food and PA. The previously published detailed methodology of the sustainable monitoring system is briefly outlined below [17,37].

#### 2.4.3. Anthropometry and Behaviours

Participants will be all children in grades two (7–8 y), four (9–10 y) and six (11–12 y) available on the day of data collection at their school who have not returned an opt-out consent form. All primary schools in the GSCRV will be invited to participate using the approach outlined below.

### 2.5. Measures/Instruments

All eligible children (grades two, four and six) in consenting schools will have height and weight measured by trained personnel. Children in grades four and six will be invited to complete, on an electronic tablet (i.e., iPad), survey instruments (Table 3) selected for suitability, reliability and validity for these age groups [17].

### 2.6. Procedures

In Term 1 of each study year (2015 baseline, 2017 and 2019) presentations will be made to school principal networks (agreed partners), with written invitations and an information pack sent to each school principal, followed up with phone calls and/or personal visits to recruit schools. Assembly and classroom presentations will be made to all children in the target grades regarding the study, at which time the Plain Language Statement and opt-out consent form will be distributed. All children in each target grade will be enrolled unless they return an opt-out consent form signed by their parents/guardians or opt out verbally. Measurements will typically be conducted over a one-day visit to each school in the second term of each year (March to June). A team of up to six trained data collectors (all with current Working with Children Checks or equivalent) will collect anthropometric and survey data in one class period. Data collectors will be trained by study leads in anthropometric measurement, accelerometer fitting, questionnaire delivery and sensitivity when engaging young children.

### 2.7. Sample Size

Based on school enrolment data, we estimate that there are 5050 children in the GSCRV in grades two, four or six across 84 primary schools in the region. Using an opt-out consent process, which delivers a >90% response rate, and assuming that 75% of schools will participate, we expect to measure more than 3000 children at each study wave meaning 3000 observations at each step and 9000 across the three study data points. BMI-z standard deviation (1.2) and intra-cluster correlation (0.027) were estimated in a previous study of >2500 Victorian school children (2014–2015). Under the stepped wedge design (ten clusters, three data collection points, five clusters) randomized to intervention at step 1, there will be an average of 300 children in each cluster, and the minimum detectable difference in BMI-z between groups with 80% power will be 0.13. This compares favourably with the observed difference achieved in previous successful intervention studies in children of 0.18, which was associated with a 3% reduction in prevalence of overweight and obesity over three years [7,8,9].

While the power for the current study is based on the stepped wedge design alone, the external parallel control group of 13 communities will add an additional comparative power to this trial.

### 2.8. Environmental Audits

Environmental audits will be conducted using a modified version of the Schools’ Environmental Audit survey [46] including elements of the international study of childhood obesity, lifestyle and the environment (ISCOLE) survey [46].

### 2.9. Ethics Approvals

Full ethics clearances have been received for all methods described above: Deakin University’s Human Research Ethics Committee (DU-HREC) 2014-279, DU-HREC 2013-095, Deakin University’s Human Ethics Advisory Group-Health (HEAG-H) HEAG-H 194_2014, HEAG-H 17 2015, HEAG-H 155_2014), the Victorian Department of Education and Training 2015_002622, 2013_002013, and the Catholic Archdiocese of Melbourne, Sale, Sandhurst and Ballarat.

## 3. Analysis Plan

Social network analysis (SNA) [47] will be used to quantify the strength and importance of relationships among people and organizations who have control over environments that affect children’s health [48]. Social network analysis software (e.g., “SNA” and “network” in R [49] will be used to calculate network statistics for each community including density and centrality and “opinion leader” positions, which are statistics used to identify people in the network who send or receive the most connections to others (the former indicating influence, the latter prominence), or who occupy other central or bridging positions in a network that are important to network diffusion and connecting relatively disconnected areas of the network. Social network dynamics will be modeled using longitudinal network models to determine how the networks change over time, the factors that predict the formation and maintenance of new relationships, and the role of the network in the diffusion information, knowledge and practice. Additional systems measurement will comprise evaluation of change in systems maps, tracking of change in subsystems and process evaluation of the communities’ responses to the systems intervention. The grounded logic models developed through this process will also allow the addition of other data sources, whether bespoke or routine data, for an understanding of the change in systems.

**Figure 2:**
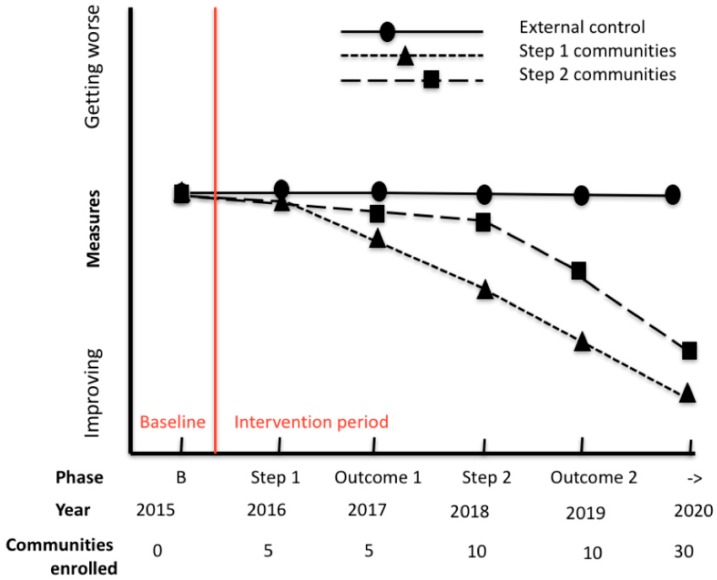
Hypothesis testing over time for the WHO STOPS trial.

The effect of the intervention on the main outcome (BMI-z) will be assessed using a linear mixed model with cluster as a random effect (community) and time (step), intervention and interaction of time by intervention as fixed effects. Because intervention enrolment will be staggered, data will be analyzed following two different approaches: (1) an intention to treat principle irrespective of when the enrolment effectively occurred; and (2) considering actual enrolment time. Secondary outcomes will be analyzed by fitting a generalized linear mixed model with link and distribution selected according to the variable (Figure 2). Missing outcomes (which we anticipate will be sparse due to the selection criteria “child present at school on the day of data collection”) will be managed using an inverse probability weighting approach.

### Economic Evaluation

The economic appraisal will assess from a “societal” and “health sector perspective” whether the intervention is value-for-money compared to current practice [50,51]. A trial-based analysis will be undertaken utilizing data collected in the trial, as will economic modelling to estimate longer-term quality of life and health sector offsets using best available information. Evaluation approaches will include cost-effectiveness analysis (CEA) focusing on obesity prevalence; cost-utility analysis (CUA) using the Child Health Utility instrument (CHU-9D) to elicit changes in participant quality of life levels; and cost-consequences analysis (CCA), which will identify other benefit considerations such as equity impacts, acceptability to stakeholders, affordability/sustainability and strength of evidence, as well as analyzing the extent of community investment in obesity-related prevention action for every dollar of public investment. A pathway approach will underpin the cost analysis, with clear specification of intervention activities and associated unit prices.

Beyond the facilitated workshops, this intervention explicitly asks each community to operationalize the intervention. This raises a number of methodological issues for the economic evaluation, such as: (i) attribution of each community’s subsequent actions to the intervention; (ii) capturing data on the extent, nature and costs of these downstream actions; (iii) attribution of the costs associated with these downstream community and household actions; and (iv) assessing which action(s) have the most positive impact. Resolution of these issues is difficult to specify in advance—there is no established practice in applying economic evaluation to a systems setting. Accordingly, we will pilot test alternative methods by conducting a retrospective economic evaluation in the project’s pilot community, which is one year post initial intervention exposure. Detailed methods for the full economic evaluation will be finalized after careful consideration of lessons learned from the pilot economic evaluation.

## 4. Conclusions

Current evidence points to systems science as the best way of identifying and addressing the complex and dynamic causes of obesity. WHO STOPS sets out to understand the ways in which the application of systems thinking could prevent childhood obesity.

## Figures and Tables

**Table 1:** Study design for the WHO STOPS cluster randomized controlled trial.

Community Type	*n*	Baseline	Step 1		Step 2	
Participant communities	5	^#^ * (0)	**^#^** (0)	^#^ * (0)	**^#^ (1)**	^#^ * (2)
	5	^#^ * (0)	**^#^ (1)**	^#^ * (2)	**^#^ (3)**	^#^ * (4)
External control	13	* (0)	(0)	* (0)	(0)	* (0)
Year		2015	2016	2017	2018	2019

**^#^** Data collection systems mapping; * Date collection individual outcomes; (X) = Years in intervention group; Data in bold denotes intervention period.

**Table 2:** Intervention design activities.

Workshop	Content
**3 h Workshop 1** Problem Identification	Background, evidence, plan presentation; fill in community capacity index; develop system logic model for “causes of childhood obesity in their community” (outcomes evidence translation, baseline measurements, base systems model).
**3 hour Workshop 2** Problem Refinement	Further evidence presentation; fill in social network analysis questionnaires; validation of system logic model on contextualised caused of childhood obesity constructed from the previous workshop (outcomes-further baseline data and knowledge translation and first validation of model).
**Half-day Workshop** Solution Formulation and Prioritisation	Steering group recruits between 50 and 200 champions from across the community who validate the systems logia model and identify priority actions for each sub-system (e.g., water system, school system, food system) related to them (outcomes; wider community validation of model and action plans).
**2 h Workshop 2** Solution Integration	Review the consolidated priority actions (outcomes; translate to institutional action plans).
**1–2 h EvaluationSessions** Adaptive Solutions	Steering: six monthly sessions to identify subsystem changes and modifications to the systems map (outcomes such as individual child measures, process change; follow up system measures).

**Table 3:** Primary and secondary outcomes of interests and proposed instruments.

Item	Outcome(s) of Interest	Instrument/Measure
Anthropometry	Body Mass Index-z score	Height and weight
	Overweight and obesity prevalence [37,38]	
Physical activity and sedentary behavior	Minutes per day (min·d^−1^) spent in moderate-to-vigorous physical activity and sedentary behavior	Modified Core Indicators and Measures of Youth Health [39] and School Health Action, Planning and Evaluation System [40] Accelerometer (sub sample)
	Proportion of participants meeting the national physical activity guidelines and screen-time recommendations [41]	
Diet Type, frequency	Usual serves of: fruit and vegetable daily	Modified version of the Simple Dietary Questionnaire [42]
	Usual frequency of non-core foods (e.g., take-away food, chips, lollies, chocolate)	
	Usual frequency of sugar-sweetened beverages	
	Proportion of participants meeting the Australian Dietary guidelines for fruit and vegetable intakes [43]	
Quality of life	Global summary score	Paediatric Quality of Life Inventory 4.0 (PedsQL)^TM^ [44] CHU-9D Child Health Utility Index [45]
	Physiological health summary score	
	Physical health summary score	
	Child Health Utility Index (CHU-9D )	
Environments	School healthy eating and activity policies and practices	Modified version of the Be Active Eat Well Environment audit [38], the International study of childhood obesity, lifestyle and the environment tool [46]
	Adherence to physical education and sport education mandate for Victorian primary schools	
Social networks (ecological data)	Characteristics of Community Leader social networks	Validated measures of socio-centric and ego-centric communication and collaboration networks
	Density, diffusion, change dynamics, key players	

## Abbreviations

The following abbreviations are used in this manuscript:

ABM	Agent-Based Model
ACTRN	Australian New Zealand Clinical Trials
BAEW	Be Active Eat Well
BMI	Body Mass Index
BMI-z	Body Mass Index z score
DES	Discrete Event Simulation
DU-HREC	Deakin University’s Human Research Ethics Committee
GLOBE	Global Obesity Centre
GMB	Group Model Building
GSCRV	Great South Coast Region of Victoria
HEAG-H	Deakin University’s Human Ethics Advisory Group-Health
ISCOLE	International Study of Childhood Obesity, Lifestyle and the Environment
SD	System Dynamics
SNA	Social Network Snalysis
WHO	World Health Organization
STOPS	Whole of Systems Trial of Prevention Strategies
